# Music in Mood Regulation and Coping Orientations in Response to COVID-19 Lockdown Measures Within the United Kingdom

**DOI:** 10.3389/fpsyg.2021.647879

**Published:** 2021-05-19

**Authors:** Noah Henry, Diana Kayser, Hauke Egermann

**Affiliations:** York Music Psychology Group, Department of Music, University of York, York, United Kingdom

**Keywords:** music, mood regulation, coping, COVID-19, lockdown

## Abstract

Music is a tool used in daily life in order to mitigate negative and enhance positive emotions. Listeners may orientate their engagement with music around its ability to facilitate particular emotional responses and to subsequently regulate mood. Existing scales have aimed to gauge both individual coping orientations in response to stress, as well as individual use of music for the purposes of mood regulation. This study utilised pre-validated scales through an online survey (*N* = 233) in order to measure whether music’s use in mood regulation is influenced by coping orientations and/or demographic variables in response to the lockdown measures imposed in the United Kingdom, as a consequence of the COVID-19 pandemic. Whilst factor analyses show that the existing theoretical structure of the COPE model has indicated a poor fit for clustered coping orientations, a subsequent five-factor structure was determined for coping orientations in response to lockdown. Analyses include observations that *positive reframing* and *active coping* (*Positive Outlook*) were strong predictors of music use in mood regulation amongst listener’s coping strategies, as was *Substance Use*. Higher *Age* indicated having a negative effect on music’s use in mood regulation, whilst factors such as gender were not seen to be significant in relation to the use of music in mood regulation within this context. These results provide insight into how individuals have engaged with music orientated coping strategies in response to a unique stressor.

## Introduction

Although many studies have aimed to measure responses to stresses amongst individuals, few have had such an opportunity to measure these stresses during a specified and ubiquitous context, such as that of a global health crisis. In the attempt to observe possible associations between the use of music in mood regulation and individual coping orientations, this research aims to expand existing knowledge on human interaction with stresses; and subsequent methods by which individuals attempt to mitigate the emotional or psychological responses to such stresses. Music has the ability to induce strong emotional responses in order to alleviate stress and negative emotions caused by some of the troubling experiences that we may encounter in our lives (Roy et al., 2009). One such stressful experience, that has become universal, is that of the 2020 COVID-19 pandemic. This particular crisis has led to the enforcement of social distancing measures on every inhabited continent, which has meant that individuals have been unable to socialise as they would under normal circumstances. Initial lockdown measures in the United Kingdom were publicly announced by Prime Minister Boris Johnson on the 23rd March 2020 (BBC News, 2020b). The measures that were included only allowed residents of the United Kingdom to leave their homes for one of four reasons: shopping for basic essentials (as infrequently as possible), one form of daily exercise, medical emergencies and travelling to or from work (but only where absolutely necessary). These measures meant individuals had to work from home, whilst households were not permitted to mix, and children were not to attend school. Measures were initially eased on 13th May 2020 in England (BBC News, 2020a).

Coping strategies occur in response to stressors and constitute a variety of methods that include individual orientations focused on solving the problem at hand through *problem-focused coping*; and mediating the emotional distress or discomfort through *emotion-focused coping* (Carver et al., 1989). Music has been observed to be an effective mood regulator and holds the ability to alter, generate, maintain or enhance emotions and moods in daily life for personal benefit and direct coping (van Goethem and Sloboda, 2011). Scales have been developed that aim to accurately quantify and measure the use of music for the purposes of mood regulation (e.g., Saarikallio, 2008) as well as coping strategies exhibited by individuals (e.g., Carver et al., 1989). We aim to test whether existing scales are applicable to the context of a global health-crisis and explore whether coping orientations and socio-demographic variables may influence the extent to which music is used as a mood regulator in response to a stressor. We hope this research will contribute to the understanding of listening behaviour and functions with specific reference to coping during times of crisis, using the COVID-19 pandemic as a context.

### Functions of Music Listening and Mood Regulation

Literature has extensively covered the functions of music listening in daily life, as well as the underlying psychological motivations behind this. ‘Felt’ emotions are experienced in response to music listening and this can produce both psychological and psychophysiological effects (Krumhansl, 1997). van Goethem and Sloboda (2011) observe the effects of ‘affect regulation.’ They find that music enables particular functions such as distraction, introspection and active coping, whilst also creating happiness and relaxation along with being an effective overall regulation device. Reports include a feeling of “having left the present world” for example, suggestive of some of the strong psychological responses that music can create (van Goethem and Sloboda, 2011, p. 216).

However, music does not necessarily generate strong psychological responses on its own, but rather within the contextual parameters of a particular situation or circumstance. Konečni (1982) argues that music listening does not take place in a vacuum, but rather in a context relative to individual scenarios. Therefore, music’s purpose is malleable according to the aim of the individual listener in response to their situation. This may be a result of factors such as cognitive processing ability; music that is not especially complicated may be preferable when undertaking complex tasks due to the choice of music being less likely to distract the listener from the task at hand, for example (Konečni, 1982). More recently, Greb et al. (2018) discuss specific situational influences to have an effect on how and why individuals engage with music. They measure particular influences that determine various functions of music listening and conclude that both individual and situational variables determine our interactions with music. The implication of this is that the methods and reasons behind why people engage with music when they do is, to some degree, dependent on the situation in which they are listening.

One of the most important situational influences on music selection appears to be an individual’s particular mood. Many studies have observed how and whether music has an effect on our moods and as such has resulted in both interesting findings and subsequent research models. Thayer et al. (1994) for example, found that music listening was effective in alleviating bad mood, raising energy and reducing tension. Music listening for the purposes of mood regulation occurs regularly and in everyday life for many people (Stewart et al., 2019). Music’s application to regulate mood can be intentional insofar as listeners may select and use music to generate desired regulatory strategies (Cook et al., 2017). Both qualitative and quantitative research has explored the processes and impressions of music as a mood regulator. Saarikallio and Erkkilä (2007) defined regulatory goals and strategies through interview data. This approach was able to discern that mood improvement was an important regulatory aim of listeners, and that the desire for mood improvement and its positive effects were comparable to hedonic motivations, recognised as a major goal for mood regulation more broadly (Saarikallio and Erkkilä, 2007). Interestingly, it was observed that music mood regulation was, on occasion, maintaining or enhancing negative moods temporarily. This was theorised to be an example of Larsen’s (2000) delayed hedonic gratification; whereby certain activities do not immediately improve mood, but rather promote positive emotions over a longer or sustained period of time. An example of this may be reflection on a negative experience when listening to sad music, which may increase melancholy or negative emotions in the short-term but increase happiness and well-being in the long-term by enabling the listener to “gain understanding and clarification of the experience” (Saarikallio and Erkkilä, 2007, p. 101). In general, music listening may be orientated toward the improvement, maintaining or intensification of mood; any of which may occur in a particular listening episode (Stewart et al., 2019). It has been suggested that music listening intentions and outcomes are mediated by levels self-awareness and “insight into the mood regulation processes occurring during music listening” (Stewart et al., 2019, p. 1). Subsequently, researchers have proposed that music has the ability to serve as a method for ‘self-therapy’ (Saarikallio, 2008).

Saarikallio and Erkkilä (2007) find that a primary goal for music as a mood regulator is that of mood control which “reflects the need for self-determination of personal mood states, the ability to voluntarily experience preferred moods and aim to achieve them” (p. 102). This process is in keeping with the control (or system) theory of mood regulation. The theory observes mood regulation as an attempt to reduce or minimise disparities between the current and desired mood states of an individual (Larsen, 2000). This is considered to be an important concept in that it relates to self-determination; a concept considered essential in self-regulation as it facilitates the experiencing of personal emotions in an unforced and voluntary manner; which in turn relates to feelings of self-autonomy. This is not limited to individual listeners, however. Musical preferences may contribute to social identity, particularly in younger listeners, which is shaped by socialisation with peers and social cognitions, both of which are considered meaningful and complementary psychologically (Miranda and Claes, 2009). This indicates that the use of music in mood regulation is not always limited to enhancing positive effects. In addition to the enhancement of mood, music may also be used specifically in order to help individuals cope with stressful situations by providing comfort individually as well as holding subsequent social or group identities (Miranda and Claes, 2009).

In sum, the use of music in mood regulation has been observed to have both individual and social applications. Individually there is internal mood alleviation and improvement according to personal desired aims; meanwhile social applications may include shared social identity and understanding that listeners may find comforting through feelings of shared identity. Quantitative methodologies have been developed to quantify the use of music as a mood regulator. The Music in Mood Regulation (MMR) scale, developed by Saarikallio (2008), is an index of seven primary factors that were observed to motivate the use of music as a mood regulator. These regulatory strategies are:

1. *Entertainment* – The creation of a positive atmosphere and happy feelings in order to sustain or enhance an existing positive mood.
2. *Revival* – The gaining of new energy from music listening when tired or stressed through renewal and relaxation.
3. *Strong sensation* – The seeking of an intense emotional experience.
4. *Diversion* – Distraction from unwanted thoughts or feelings through music use.
5. *Discharge* – The releasing, or venting, of sadness or anger though music that expresses such emotions.
6. *Mental work* – The involvement of music as a framework by which contemplation and reappraisal of emotional preoccupations are possible.
7. *Solace* – The seeking of personal feelings to be accepted and understood when feeling sad or troubled.

Overall, qualitative research has been synthesised into quantitative scales capable of measuring how music is used to regulate mood and in turn this model and its approach form the basis for the music regulatory part of this study. To date, research into the use of music as a mood regulator has largely focused on music mood regulation during adolescence (Saarikallio and Erkkilä, 2007; Miranda and Claes, 2009) and in response to diagnosed health issues (Silverman, 2020). Similar effects have also been observed in adult populations insofar as adult’s emotional experiences can too be intensified by music listening (Karreman et al., 2017). Emotion-based regulatory strategies of music have additionally been reported at different stages of life, from adolescence to old age, suggestive of music’s long-term placement in assisting emotional regulation (Saarikallio, 2010). At large, the unprecedented situation in which this study is conducted will provide unique insight into how music may be used as a mood regulator across a broader variety of age groups in response to a stressor with unique applicability to the general population.

### Coping in Response to Stress

Existing research has described music’s function in daily life as a tool through which to manage stressful situations or experiences. The suggestive use of music as escapism is implicit of its use as a means by which to cope with such situations. van Goethem and Sloboda’s (2011) explicit mentioning of *active coping* is of particular importance to this study insofar as this is often described as a primary coping strategy that is used by individuals in response to stresses. There has been extensive psychological research conducted into coping strategies, as well as an established series of scales that aim to measure these strategies. The Coping Orientations to Problems Experienced (COPE) inventory, for example, has been used extensively to establish individuals’ personal coping strategies or orientations based on a series of responses compiled by Carver et al. (1989). Strategies such as *active coping*; along with *self-distraction*, *acceptance* and *positive reframing*, formulate two primary branches of coping strategies: *problem-focused coping* and *emotion-focused coping* (Carver et al., 1989).

In their identifying of these two branches of coping strategies, Lazarus and Folkman (1984) define *problem-focused coping* as consisting of efforts to modify the problem at hand, often by generating solutions and weighing up the pros and cons of these different options. Alternatively, *emotion-focused coping* is defined as the aim to mitigate emotional distress caused by, or associated, with the problem (Lazarus and Folkman, 1984). The range of *emotion-focused* coping strategies is particularly broad and may include denial, focusing on and venting of emotions, positive reinterpretation of events and the seeking out of social support (Baker and Berenbaum, 2007). Although *emotion-focused* coping strategies are related to reappraisal (the process of assessing situations in different manners) by leading to changes in the way experiences are construed, Lazarus and Folkman (1984) discern an important differentiation between *emotion-focused* coping strategies and reappraisal itself. They assert that not all *emotion-focused* coping strategies change the meaning of events directly and are not synonymous with reappraisals in general. In other words, some, but not all, *emotion-focused* coping strategies are reappraisals (see Lazarus and Folkman, 1984).

In addition to the more widely established *emotion-focused* and *problem-focused* coping strategies, *dysfunctional coping* has been discussed as a potential third set of coping strategies. Carver et al. (1989) “measure coping responses that are arguably less useful” than *problem-focused* or *emotion-focused* coping strategies (p. 267). These *dysfunctional coping* strategies are not considered part of the other two primary branches that were previously mentioned, but rather formulate a variety of coping responses that may be viewed as being less effective in coping with stressful situations. These strategies may include *behavioural disengagement*, *denial* and *venting* for instance (Coolidge et al., 2000). Miranda and Claes (2009) find that avoidance or disengagement coping by music listening is a passive strategy that aims to distract the individual from the problem and related stress, rather than to mitigate its effects. Furthermore, the recurring use of this tactic is associated with more symptoms of emotional instability over time, separating it from *emotion-focused* coping strategies by not ultimately improving the individual’s experience (Miranda and Claes, 2009). In other words, *dysfunctional* coping strategies should be deemed separate from *emotion-focused* coping strategies because, although there may be emotional elements through *venting* for instance, this coping strategy does not improve the overall emotional state of an individual over time.

There have been various methods used to measure both *problem-focused* and *emotion-focused* coping strategies. These include the Ways of Coping (Lazarus and Folkman, 1984; Folkman and Lazarus, 1985), the Multidimensional Coping Inventory (Endler and Parker, 1990), the Coping Strategies Inventory (Tobin et al., 1989) as well as the previously mentioned Coping Orientation to Problems Experienced, or COPE, inventory (Carver et al., 1989). Each of these have differences in how they measure coping strategies; however, they all assess *problem-focused* coping strategies as well as strategies that give attention to the emotional implications of the situation rather than the stressor, i.e., *emotion-focused* coping strategies (Carver, 1997).

A consideration to make when it comes to the use and measurement of coping strategies, however, is whether stable coping ‘styles’ exist. This issue has seemed divisive amongst sources and it seeks to determine whether individuals approach stresses with an established set of coping strategies rather than dealing with each stressful context in a new way (Carver et al., 1989). The alternate position to this is rather that coping strategies are dynamic, and contextual to the situation that individuals find themselves in Carver et al. (1989). It had been argued that particular personality traits may predispose responses to stresses and subsequent coping strategies, however, this was concluded to be a null finding in subsequent studies, with it being likely that traditional personality dispositions are not useful predictors of coping strategies (Folkman and Lazarus, 1980; Carver et al., 1989). On the other hand, many researchers are not keen to assume that personality traits play no role in determining an individual’s coping strategy in response to stressful situations.

This is where the COPE model demonstrates itself as a useful method with which to measure individual coping strategies insofar as Carver et al. (1989) utilise statements that pertain to both coping dispositions as well as situational coping responses. “In developing our coping inventory, we made an effort to include only items that could be answered from both orientations, so that the inventory could be used to examine both coping dispositions and situation-specific coping tendencies (depending on the researcher’s needs and desires)” (Carver et al., 1989, pp. 270–271). In short, the COPE scale incorporates statements in relation to what a person actually did, or is currently doing, in a specific coping episode.

However, a limitation with Carver et al.’s (1989) subsequent inventory was that it was often considered by participants to be too long (Carver, 1997). Carver (1997) subsequently developed a reduced, or brief, version of the COPE inventory in order to make it a more practical research tool. Both the full and brief COPE models infer and measure a set of 14 subscales each addressed by two items, that either fall into *emotion-focused* coping strategies, *problem focused* coping strategies or *dysfunctional* coping strategies (Cooper et al., 2006). These 14 subscales are shown in Table 1.

**TABLE 1:** COPE strategies by subgroup.

Emotion-focused coping strategies	*Acceptance* – The acknowledgement or accepting of reality of the situation.
	*Using Emotional Support* – The seeking of moral support, sympathy and emotional understanding.
	*Humour* – The minimising of the stressor’s significance or seriousness by making light of it.
	*Positive Reframing* – The process by which the circumstances are reframed in one’s mind in order to see the potential positives of the situation.
	*Religion* – The seeking of emotional support or understanding of the stressor through prayer or mediation within a particular faith.
Problem-focused coping strategies	*Active Coping* – The process of taking proactive steps in an attempt to remove or circumvent the stressor and alleviate its effects.
	*Planning* – The consideration of exactly how to cope with the stressor by developing action strategies in order to determine what steps to take in order to deal with the problem.
	*Using Instrumental Support* – The seeking of advice, assistance or information with regards to the stressor.
Dysfunctional coping strategies	*Behavioural Disengagement* – Minimising or even giving up on attempts to deal with the stressor.
	*Denial* – The refusal to acknowledge the stressor’s occurrence or full implications.
	*Self-Distraction* – Disengagement from the stressor by focusing on other activities to take one’s mind off the problem.
	*Self-Blame* – The process of determining personal mistakes that could have otherwise reduced the stressor’s significance.
	*Substance Use* – The turning to drugs or alcohol in order to suppress the process of giving attention to, and dealing with, the stressor.
	*Venting* – The act of focusing on, and venting of, emotions in relation to the stressor.

*Adapted from Coolidge et al. (2000).*

### Music and Coping

Direct links have been made between music listening in functioning as both an *emotion-focused* and *problem-focused* coping strategy. Miranda and Claes (2009) found that adolescents use music listening in *emotion-focused* manners in order to regulate emotions and in *problem-focused* manners in order to reflect on solutions to stressful issues via a self-report questionnaire. Miranda et al. (2010) report that adolescents may use music to mediate neuroticism; especially when scoring low on avoidance, or disengagement coping styles by music listening. It was found that *problem-focused* coping by music listening may moderately reduce neuroticism, which often induces feelings such as worry, fear, anxiety and depressed or low moods which music was able to alleviate to some extent (Miranda et al., 2010). In other words, when the problem at hand was low or negative mood inducing, music listening served as a *problem-focused* coping strategy, much like Carver et al.’s (1989) definitions *active coping* and *planning*, by functioning as a direct solution to the problem at hand, general low mood in this example. This is suggestive that the strategies by which individuals cope with stressful situations influence their potential application of music in response to stressors. When it came to adults, Karreman et al. (2017) found that neuroticism was a contributing factor in explaining inter-individual differences in the effects of emotion regulation strategies. However, it is subsequently suggested that individuals experience or seek to experience emotional extremes when they are at younger stages of adulthood (Karreman et al., 2017).

van Goethem and Sloboda’s (2011) description of music listening as an *active coping* strategy furthers this notion. Carver et al. (1989) define *active coping* to be a strategy through which individuals take proactive steps to mitigate the negative effects of a stressor. van Goethem and Sloboda (2011) argue that active coping with regards to music appears to occur when individuals are engaging in other activities. They subsequently theorise that active coping does not appear in Saarikallio and Erkkilä’s (2007) qualitative list of music mood regulatory strategies due to their particular study not prompting participants to consider specific listening episodes. This relates back to the earlier point made by Konečni (1982), in which it is argued that music listening takes place within particular listening contexts that contribute to the overall experience and what subsequent effect music will have.

Furthermore, Ter Bogt et al. (2010) formulate a typology of music listeners, subsequently suggesting that different groups of listeners have varying extents to which music is involved in mood enhancement, coping and personal and social identity. Three groups were identified: High-Involved listeners, who “experienced music as a very important medium and used music most often for mood enhancement”; and Medium- and Low-Involved listeners who, whilst forming two separate groups, had less intense importance of music in mood enhancement (Ter Bogt et al., 2010, p. 147). The findings of this study were that High-Involved listeners experienced the most intense positive effects when listening to music, whilst both High- and Medium-Involved listeners both reported more negative effects (such as anger and sadness) compared to the Low-Involved listeners. However, the Low-Involved group listened to music frequently and used it as a mood enhancer nevertheless (Ter Bogt et al., 2010). In summary, individuals that are the most strongly “moved by music” either positively or negatively, use it for mood enhancement and coping more often (Ter Bogt et al., 2010, p. 147). From this, it is plausible to consider that those responding to higher use of music in mood regulation are more likely to benefit the most from its ability to enhance moods and emotion. Within the context of lockdown situations during the COVID-19 pandemic, it has been indicated that music related activities have increased through listening, singing, dancing or playing instruments, and that this has been accompanied by efforts to cope with the stressor at hand (Cabedo-Mas et al., 2021). Additionally, Krause et al. (2021) found that life satisfaction within students was higher than normal when individuals increased the time they spent listening to music, suggesting an association between the amount of time spent listening and well-being.

Therefore, from the strategies laid out in the coping psychology literature, it may seem that those using more *emotion-focused* coping strategies are more likely to benefit and therefore use music as a mood regulator in response to stress. It has also been theorised, however, that negative or stressful life events can lead otherwise emotionally stable individuals to seek refuge in recurrent coping by music listening (Miranda et al., 2010), and the music can indeed lead to unhealthy coping habits (Silverman, 2020). This may be reflected in individuals effected by lockdown most through abrupt changes, such as being left unemployed or placed on furloughed status. Consequently, it is worth considering whether existing scales are applicable to the specified context of this study. Silverman (2020) found that when applying the Brief COPE scale to the Brief MMR scale, music could be used as a coping strategy to enhance wellbeing. Finding that music mood regulatory strategies were associated with subsequent coping strategies, Silverman (2020) argues that music-based affect regulation may be orientated to augment the likelihood of recovery in adults with mental health conditions. Whilst interesting, the orientation of this study differs from ours insofar as music’s use was expressed as an independent variable, with coping strategies acting as dependent variables. However, other research into coping orientations has viewed them as being independent variables or predictors that may inform subsequent behaviour (e.g., Carver et al., 1989; Corbin et al., 2013; Gustems-Carnicer and Calderón, 2013).

In summary, coping orientations may be stable dispositions relative to each individual, and that these sets of strategies have been engaged in response to the lockdown measures brought about by the COVID-19 pandemic. Music has been observed to be used in response to stressors in order to mitigate negative effects and enhance positive effects. These effects have been quantified and measured in validated scales, such as the MMR inventory (Saarikallio, 2008). Cumulatively, consistent engagement with music may increase its prevalence as a mood regulator amongst individual listeners. As such, those reporting higher frequencies in music listening may additionally report higher use of music for the purposes of regulating mood to enhance, maintain or attain positive moods. Furthermore, music listening may become a recurrent coping strategy when faced with significant or abrupt stressors; potentially seen in those most heavily affected by lockdown consequences of the COVID-19 pandemic (such as being unemployed or placed on furlough).

Extant research has indicated that the ways in which individuals cope with stressors and use music to regulate mood are associated. Music use may assist as a practical and adaptive coping strategy against stressors and can be used for ‘affect’ regulation and coping in daily life; as well as in response to tangible stressors (Miranda and Claes, 2009; Silverman, 2020). It is within these theoretical frameworks that this study aims to understand whether coping strategies and the use of music in mood regulation are related; and whether existing scales may be applicable to the context of lockdown.

## Aims and Research Questions

The aim of this study was to investigate whether there are associations between the use of music as a mood regulator and an individual’s coping strategies. This was carried out using the specific context of lockdown during the COVID-19 pandemic and was predicated on the notion that music’s properties in overall mood regulation have a relationship with coping dispositions displayed during times of stress. Existing scales have been used in order to gauge the extent to which they are applicable in this context. In addition to this, specific effects of lockdown have been measured in order to observe how music may be used in mood regulation in response to those effected most, such as those with job insecurity, as well as the household contexts in which individuals experienced lockdown.

### Research Questions

We conducted an online survey in order to explore the following research questions:

1. Is the Brief COPE model appropriate to observe coping in response to lockdown?
2. Is the MMR model appropriate to observe music mood regulation in response to lockdown?
3. Do different coping strategies and/or demographic variables influence how an individual may use music as a mood regulator in response to lockdown?

## Materials and Methods

### Participants

The total number of respondents to this survey was 233 (44.6% male, 54.1% female, 0.4% other and 0.9% prefer not to say) between the ages of 18 and 80 years (*M* = 41.33, *SD* = 15.04). The sample consisted of a broad variety of individuals, of which 97 (41.6%) stated they were either amateur, higher level or professional musicians. The lockdown and employment statuses of the sample are shown in Tables 2, 3. Respondents were gathered by a shareable Internet link distributed through email lists at the University of York, over social media and word of mouth. All respondents were required to be 18 or over and residents of the United Kingdom, which required confirmation before participating. Although the requirement of all respondents to be United Kingdom residents may seem to raise the prospect of regionalist bias; this was necessary due the parameters of dates that were used in this study. It was deemed important that we reduce random variations in data by not having respondents who may have had different experiences of lockdown within the specified dates, depending on their own region’s lockdown phase. Ethical approval was given by the Arts and Humanities Ethics Committee at the University of York data was collected between 26th June and 28th July 2020.

**TABLE 2:** Lockdown status of respondents.

Lockdown status	Frequency	Percent	Age		Gender	
			Mean	SD	Male	Female
Alone	29	12.40	44.59	13.752	13	16
Within a family unit	122	52.40	40.36	14.572	56	64
With one other person	60	25.80	44.57	15.408	27	32
Within a house-share	17	7.30	32.35	14.765	7	10
Other	5	2.10	38.00	19.621	1	4
Total	233	100	41.33	15.043	104	126

*Values in gender common report frequency. Gender information was not provided by three participants, hence N = 230 within male and female categories.*

**TABLE 3:** Employment status of respondents.

Employment status	Frequency	Percent	Age		Gender	
			Mean	SD	Male	Female
Employed full-time and able to work	105	45.10	43.01	12.193	52	52
Employed part-time and able to work	28	12.00	39.36	13.458	5	23
Furloughed or otherwise unable to work (but still employed)	30	12.90	32.43	14.607	15	14
Unemployed	19	8.20	28.11	13.362	6	13
Retired	21	9.00	63.10	6.09	15	5
Other	30	12.90	39.37	14.187	11	19
Total	233	100	41.33	15.043	104	126

*Values in gender common represent frequency. Gender information was not provided by three participants, hence N = 230 within male and female categories.*

### Materials and Procedure

The material used for this study was an online survey, made and distributed using the Qualtrics software (Supplementary Appendix A). The survey consisted of primary information regarding the study as well as the requirement for informed consent before proceeding beyond the opening page. This survey included a link to a participant information sheet which provided further details on ethics and data management. In addition to this, both the information sheet and final page of the survey included links to appropriate mental health and wellbeing services, should any respondents have felt stress or discomfort regarding the survey.

Furthermore, the time scale of the study was made clear to participants insofar that this study aims to measure its parameters within the specific context of the United Kingdom’s most restricted lockdown period. This was defined by using the British government’s imposition of lockdown measures on 23rd March 2020, to the initial easing of measures on 13th May 2020. This time bracket was made clear to respondents before proceeding with the survey using the following phrasing:

“*This survey will ask questions about how you have been using music during lockdown. In this research, we are defining the lockdown period according to the British government’s imposing of restrictions from the 23rd March 2020, to the initial easing of restrictions on the 13th May 2020. Therefore, it is this period from the 23rd March–13th May that should be the context from which you consider responses*.”

### Measures

#### Accessing Music

These measures asked how many hours each day respondents had listened to music on average, whether this was more, less or the same as before lockdown and through what media they had primarily been listening.

#### Music in Mood Regulation (MMR) Scale

The MMR research model developed by Saarikallio (2008) is a 40-item inventory intended to measure seven separate parameters that indicate the use of music as a mood regulator. This inventory was used in full, with questions reframed to the specific context of lockdown during the COVID-19 crisis in the United Kingdom. This scale is measured on a five-point Likert scale rated from *Strongly Disagree* to *Strongly Agree*.

#### Brief COPE Scale

The brief COPE scale is a condensed version of the original one developed by Carver et al. (1989). Carver (1997) developed this smaller inventory based on feedback that the original model was too long. As such, this 28-item inventory seems more pertinent for the purpose of this study and measures the degree to which individuals use 14 coping strategies in response to stresses. Like the MMR model, statements were reframed according to the context of the study. In addition to this, this study reduced the scale on which this model measures data from four increments, to 3 which were scaled from 1 (“*I haven’t been doing this at all*”) to 3 (“*I have been doing this a lot*”), with 2 (“*I have been doing this some of the time*”) as the intermittent value.

#### Lockdown Status

These measures included the living context of an individual’s lockdown which included living alone, within a family unit or a house-share. A further measure was employment status, which included options such as: employed full-time and able to work, employed part-time and able to work, furloughed or otherwise unable to work (but still employed), unemployed and retired. These measures also included a five-point Likert scale on which respondents were able to rate the degree to which they felt they accurately remembered their engagement with music under lockdown. This was necessary to account for, given the retrospective nature of this study.

#### Demographics

Demographic measures included age, gender, musical background (non-musician, amateur musician or professional musician) and attained level of education.

### Statistical Approach

Our approach to statistical analysis was predicated on measuring associations between pre-validated scales on how individuals cope with stressors, and the extent to which they use music for mood regulation purposes. To do this, we first needed to identify a factor structure within each model separately, in order to observe whether they were applicable to the context given. This would subsequently allow us to observe associations between the two models and was conducted through factor analyses. This was to address our first research question. Next, we would observe correlations between the time spent listening to music and the MMR scale to further validate music listening with the use of music in mood regulation. To answer our third question, we conducted a MANOVA, with identified coping strategies and other independent variables as predictors of music in mood regulation.

## Results

### Factor Analysis of COPE

A Confirmatory Factor Analysis (CFA) was carried out on the items of the COPE model in JASP (version 0.13.1). This indicated a poor fit between the clustered groups of the research model (*Emotion-Focused Coping*, *Problem-Focused Coping*, and *Dysfunctional Coping*) and the observed data [χ^2^(347) = 1528.791, *p* < 0.001, CFI = 0.442, TLI = 0.392, RMSEA = 0.121]. It was therefore necessary to conduct an Exploratory Factor Analysis (EFA) into the COPE model, in order to determine the factor structure of the 28 items. The EFA was carried out in R (version 3.6.1) and an initial inspection of box plots was conducted in order to reduce outlying items where participants had exclusively answered “*I haven’t been doing this at all.*” This led to the exclusion of Items 11, 19, 20, 26, and 28 (see Supplementary Appendix A).

The EFA that was conducted used parallel analysis with an oblimin rotation and weighted least squares (WLS) estimation method in order to determine the number of factors; the fa.parallel function in psych package (version 2.0.7) was used. The WLS estimation method fits this model to polychoric correlations (Barendse et al., 2015). This showed that a 5-factor solution fits the observed data best [χ^2^(253) = 1916.85, TLI = 0.803, RMSEA = 0.074]. However, the scoring of some items was low onto these factors and as such items 4, 7, and 12 (see Supplementary Appendix A), which all scored below 0.4 on each factor, were removed and the EFA was conducted again with the remaining factors. The results of this are shown in Table 4.

**TABLE 4:** Exploratory factor analysis of COPE.

COPE item	Factor 1	Factor 2	Factor 3	Factor 4	Factor 5
*Using Instrumental Support*: Getting advice from others	**0.80**	–0.04	–0.03	–0.02	0.07
*Using Emotional Support*: Support from others	**0.76**	0.05	0.05	–0.02	–0.11
*Using Instrumental Support*: Trying to get advice from others	**0.73**	–0.02	–0.05	0.02	0.17
*Using Emotional Support*: Comfort from someone	**0.60**	0.03	0.16	0.09	–0.09
*Substance Use*: Alcohol or drugs to feel better	–0.01	**0.97**	–0.03	0.02	–0.02
*Substance Use*: Alcohol or drugs to get through it	–0.01	**0.96**	0.01	–0.01	0.04
*Positive Reframing*: Looking for something good	–0.09	–0.03	**0.69**	0.09	0.09
*Positive Reframing*: See in a different light	0.03	–0.04	**0.64**	0.02	0.08
*Active Coping*: Doing something about situation	0.16	0.12	**0.50**	–0.11	–0.17
*Active Coping*: Make situation better	0.10	–0.02	**0.49**	0.03	–0.05
*Planning*: Developing strategy	0.24	0.04	**0.41**	–0.08	–0.01
*Acceptance*: Learning to live with it	–0.08	0.00	0.38	0.09	–0.10
*Humour*: Making fun	–0.02	–0.02	0.05	**0.88**	–0.05
*Humour*: Making jokes	0.02	0.03	–0.03	**0.86**	0.05
*Self-Distraction*: Think about it less through other activities	–0.04	0.09	0.18	–0.01	**0.52**
*Venting*: Expressing negative feelings	0.32	0.01	–0.04	0.07	**0.47**
*Venting*: Saying Things to let feelings escape	0.16	0.09	–0.03	0.14	**0.45**
*Self-Blame*: Criticising myself	0.14	0.13	–0.02	–0.03	**0.42**
*Self-Distraction*: Work or activities to take mind off things	–0.05	0.04	0.20	–0.10	0.38

*The rotation used in oblimin. The loadings with λ ≥ 0.40 are highlighted in bold.*

**FIGURE 1:**
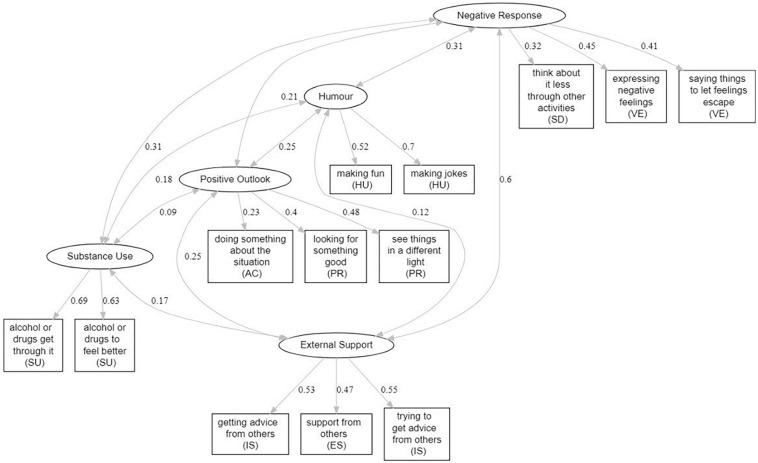
Model plot of COPE model based on CFA. Numerical values beside downward arrows indicate parameter estimates. Numerical values beside bi-directional arrows indicate covariances between factors.

The two or three highest scoring values of each of the five factors revealed that the subscales: *Using Emotional Support* and *Using Instrumental Support* (items 13, 15, and 16) loaded as Factor 1 (*External Support*), *Substance Use* (items 23 and 24) loaded as factor 2 (*Substance Use*), *Positive Reframing* and *Active Coping* (items 2, 5, and 6) formed factor 3 (*Positive Outlook*), *Humour* (items 8 and 9) loaded as factor 4 (*Humour*) whilst *Self-Distraction* and *Venting* (items 18, 21, and 22) formed factor 5 (*Negative Response*). These items were then used in a CFA, based on the factors produced by the EFA. The model was fit using the CFA function in the lavaan package (Rosseel, 2012) in R, using oblimin rotation. Factor scores are based on the WLSMV estimation method and fixing residual variances of each latent variable to 1. The CFA demonstrated a good fit between the observed data and factor structure implied by the EFA [χ^2^(55) = 80.119, *p* = 0.015, CFI = 0.956, TLI = 0.938, RMSEA = 0.044; scaled indices are reported]. Figure 1 indicates the factor structure suggested by these analyses.

### Factor Analysis of MMR Inventory

A CFA was carried out on the items of the MMR inventory which indicated that the existing model was a good fit for the observed data [χ^2^(719) = 902.143, *p* < 0.001, CFI = 0.917, TLI = 0.910, RMSEA = 0.033; scaled indices are reported]. The model was fit using the CFA function in the lavaan package (Rosseel, 2012) in R, using oblimin rotation. Factor scores are based on the WLSMV estimation method and fixing residual variances of each latent variable to 1. These factor scores have been used in subsequent analyses and can be seen in Supplementary Appendix B. The subsequent factor scores from both the COPE and MMR models were saved and extracted from R (version 3.6.1) using the Lavaan syntax and imported back into SPSS (version 26) for further analyses. A relationship between time spent listening and the MMR scale was tested through a correlation analysis in order to observe whether hours spent listening was positively correlated to the degree that listeners use music as a mood regulation strategy. This was in order to further validate the notion that MMR strategies are positively associated with music listening. These correlations are shown in Table 5.

**TABLE 5:** Bivariate correlation of hours spent listening and MMR subscales.

	Hours spent listening
*Entertainment*	0.394**
*Revival*	0.384**
*Strong Sensation*	0.320**
*Diversion*	0.353**
*Discharge*	0.244**
*Mental Work*	0.354**
*Solace*	0.358**

Extracted factor scores have been used for this analysis. **Correlation is significant at the 0.01 level (two-tailed). For all correlations p < 0.001.

This showed weak positive correlations between hours spent listening and *Entertainment*, *Revival*, *Strong Sensation*, *Diversion*, *Discharge*, *Mental Work* and *Solace*; all seven MMR subscales.

### Associations Between COPE and MMR

In order to test how and whether the subsequent factors of the research models are affected, a multivariate ANOVA was conducted; with factors of the COPE model generated by the factor analyses as predictors and the seven MMR factors as dependent variables, shown in Table 6. Additionally, we included the independent variables age, gender, lockdown status and employment status, in order to control for participant characteristics that might potentially covary with COPE factors and also influence MMR factors. Categorical variables (*Gender*, *Lockdown Status*, and *Employment Status*) were incorporated as fixed factors and *Age* and COPE factors were incorporated as covariates.

**TABLE 6:** MANOVA of predictors of MMR factor scores.

Independent variables	Pillai’s trace	*F*	Error df	*p*	$\eta_{\text{p}}^{2}$	Observed power
*External Support*	0.024	0.743	208	0.636	0.024	0.316
*Substance Use*	0.065	2.057	208	0.050*	0.065	0.785
*Positive Outlook*	0.109	3.651	208	0.001*	0.109	0.973
*Humour*	0.028	0.859	208	0.540	0.028	0.366
*Negative Response*	0.017	0.510	208	0.827	0.017	0.219
*Employment Status*	0.183	1.149	1060	0.255	0.037	0.960
*Lockdown Status*	0.142	1.107	844	0.321	0.035	0.908
*Age*	0.060	1.888	208	0.073^+^	0.060	0.743
*Gender*	0.095	0.976	630	0.491	0.032	0.764

Z standardised values were used in this analysis. *p < 0.05. ^+^indicates non-significant trend. N = 233.

Table 6 shows that several independent variables were significantly related to the multivariate analyses of MMR subscales. To make the model more parsimonious, we removed all independent variables that neither indicated significance or a non-significant trend in relation to MMR subscales. This resulted in *Gender*, *Employment Status*, *Lockdown Status*, *External Support*, *Negative Response*, and *Humour* being excluded as being predictors. We re-ran the analysis with the reduced model, the results of which can be seen in Table 7.

**TABLE 7:** MANOVA of predictors of MMR factor scores with non-significant predictors excluded.

Effect	Pillai’s trace	*F*	Error df	*p*	$\eta_{\text{p}}^{2}$	Observed power
*Substance Use*	0.090	3.161	223	0.003**	0.090	0.946
*Positive Outlook*	0.115	4.120	223	<0.001**	0.115	0.987
*Age*	0.088	3.060	223	0.004**	0.088	0.938

Z standardised values were used in this analysis. **p < 0.01. N = 233.

**FIGURE 2:**
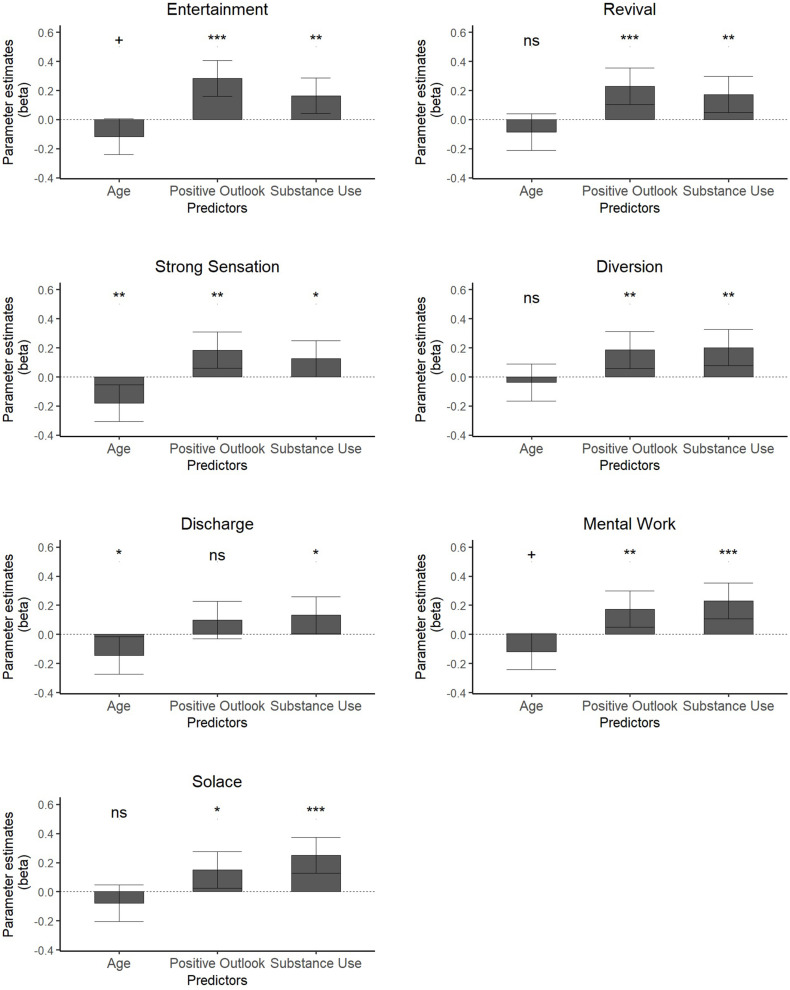
Plots of parameter estimates of independent variables and MMR factors. Error bars indicate 95% confidence Intervals. *p < 0.05, **p < 01, ***p < 0.001. ^+^indicates non-significant trend.

These results indicated that *Positive Outlook*, *Substance Use*, and *Age* were significant predictors of MMR subscales. The beta-coefficient parameter estimates were then subsequently obtained to identify the direction and strength of this association separated by each individual MMR factor. These results are presented in Table 8 and Figure 2.

**TABLE 8:** Parameter estimates of significant MMR predictors.

Dependent variable	Parameter	Beta	SE	*T*	*p*
*Entertainment*	*Positive Outlook*	0.283	0.062	4.544	<0.001***
	*Substance Use*	0.164	0.062	2.655	0.008**
	*Age*	–0.117	0.062	–1.869	0.063^+^
*Revival*	*Positive Outlook*	0.229	0.064	3.593	<0.001**
	*Substance Use*	0.172	0.063	2.737	0.007**
	*Age*	–0.086	0.064	–1.349	0.179
*Strong Sensation*	*Positive Outlook*	0.185	0.064	2.894	0.004**
	*Substance Use*	0.125	0.063	1.988	0.048*
	*Age*	–0.179	0.064	–2.802	0.006**
*Diversion*	*Positive Outlook*	0.185	0.064	2.875	0.004**
	*Substance Use*	0.202	0.064	3.174	0.002**
	*Age*	–0.038	0.064	–0.590	0.556
*Discharge*	*Positive Outlook*	0.098	0.065	1.501	0.135
	*Substance Use*	0.132	0.065	2.045	0.042*
	*Age*	–0.144	0.065	–2.201	0.029*
*Mental Work*	*Positive Outlook*	0.173	0.063	2.730	0.007**
	*Substance Use*	0.231	0.063	3.689	<0.001***
	*Age*	–0.119	0.063	–1.879	0.062^+^
*Solace*	*Positive Outlook*	0.150	0.064	2.362	0.019*
	*Substance Use*	0.250	0.063	3.968	<0.001***
	*Age*	–0.079	0.064	–1.241	0.216

Z standardised values were used in this analysis. *p < 0.05. **p < 0.01, ***p < 0.001. ^+^indicates non-significant trend. N = 233.

The analyses indicated that *Substance Use* had a positive effect on each MMR subscale, whilst *Positive Outlook* had a positive effect on each subscale except *Discharge*. *Age* indicated having negative effects on *Strong Sensation* and *Discharge*. Additionally, *Age* indicated non-significant trends in the cases of *Entertainment* and *Mental Work*. Again, these appeared to be negative associations.

With regards to the identified COPE factors, *Positive Outlook* appeared to be a stronger predictor of the MMR subscales *Entertainment*, *Revival* and *Strong Sensation* than *Substance Use*, as suggested by the parameter estimates. Meanwhile, *Substance Use* was a stronger predictor of *Diversion, Discharge*, *Mental Work* and *Solace* than *Positive Outlook*.

*Age* was a stronger negative predictor of *Strong Sensation* and *Discharge* than *Substance Use* was a positive predictor, however, *Positive Outlook* was indicated as a stronger positive predictor of *Strong Sensation*. These results indicate that two of the identified COPE factors were significant predictors of the use of music in mood regulation, and that this association was in a positive direction. *Age* indicated negative associations in relation to two MMR subscales, however, did not appear to be a negative predictor of music in mood regulation as a whole.

## Discussion

This study investigated coping strategies and the use of music as a mood regulator in response to lockdown measures within the United Kingdom. The aim was to observe whether existing scales are appropriate to measure coping orientations and regulatory focused engagement with music under these circumstances. Ultimately, the focus was to measure how music may be used in order to help individuals mitigate the negative effects of the stressor, and whether this may be predicted by individual coping orientations or strategies.

### COPE Scale’s Applicability to Lockdown

A CFA indicated that the COPE scale (Carver et al., 1989) was not an appropriate fit for observed data when subscales were clustered into the theoretical clustering of e*motion-focused coping*, *problem-focused coping* and *dysfunctional coping*. Rather, an EFA produced a five-factor solution that resulted in the subscales *Use of Emotional Support* and *Use of Instrumental Support* as Factor 1 (*External Support*), *Substance Use* as Factor 2, *Positive Reframing* and *Active Coping* as Factor 3 (*Positive Outlook*), *Humour* as Factor 4 and *Self-Distraction* and *Venting* as Factor 5 (*Negative Response*). The outcome of the factor analyses indicate that the COPE model may not apply to the context of lockdown when analysed according to the clustering of *problem-focused*, *emotion-focused* and *dysfunctional* coping strategies. Rather, this may suggest that coping styles and strategies are situation specific due to the intercorrelation of COPE subscales outside of clustered groups; as shown by the formation of *Using Emotional Support* and *Using Instrumental Support* as a single factor, for example.

### MMR Scale’s Applicability to Lockdown

A CFA of the MMR scale (Saarikallio, 2008) indicated that the theoretical model was a good fit for the observed data and as such, an EFA was not necessary for this model. The use of music as a mood regulator was further observed to be positively correlated with time spent listening, which was the case for each MMR subscale. This further indicates a general trend that increasing the amount of time spent listening to music results in its function as a mood regulator becoming more likely for each subscale. This validates the assumption that engagement with the use of music as a mood regulator is positively associated with higher or more pro-active engagement with music. However, although these correlations were all highly significant (*p* < 0.001 for each MMR subscale) they were nevertheless weak. Future research should consider whether time spent listening increases the likelihood of music in mood regulation more in depth. It would especially be worth exploring whether the amount of time spent listening to music merely increases the likelihood of each MMR subscale being used, or whether individuals that already use music in regulatory manners therefore listen to music more as they get more from it in the ways of mood regulation. It has been suggested by some recently published studies that higher levels of music listening during lockdown has had beneficial effects in increasing life-satisfaction and that it has been effective in helping people to cope (Cabedo-Mas et al., 2021; Krause et al., 2021). Whilst this suggests that higher levels of engagement with music during lockdown is effective in mitigating some of the most negative effects of the pandemic, the direction of this relationship is not inherently clear from this study and should be studied further.

### Predictors of MMR Subscales

An initial MANOVA controlled for all independent variables and their effect on MMR subscales. These independent variables included the five COPE factors identified through the factor analyses (*External Support, Substance Use*, *Positive Outlook*, *Humour*, and *Negative Response*) and demographic variables (*Age*, *Gender*, *Employment Status*, and *Lockdown Status*). From this analysis, *Substance Use* and *Positive Outlook* indicated significance in relation to MMR subscales, whilst *Age* indicated a non-significant trend in relation to subscales. None of the other independent variables indicated associations with MMR subscales. We therefore excluded these variables and re-ran the analysis to make the model more parsimonious.

The subsequent MANOVA with reduced predictors indicated that *Substance Use* was positively associated with each MMR subscale, whilst *Positive Outlook* was positively associated with each subscale except *Discharge*. *Age* was a negative predictor of *Strong Sensation* and *Discharge* and indicated non-significant trends in relation to *Entertainment* and *Mental Work*.

This suggests that particular coping responses may be able to predict the methods by which individuals use music in response to stress; with specific regards to the lockdown measures imposed due to the COVID-19 pandemic. *Positive Outlook* (a method by which individuals have responded to the pandemic through *Positive Reframing* and *Active Coping*) has been observed to be a significant predictor of the use of music as a mood regulator except for regulation through *Discharge*. This supports existing theories and hypotheses that music’s use in mood regulation may be to raise or enhance positive effects or emotion in general.

Interestingly, with *Substance Use* serving as a positive predictor of each MMR subscale, it suggests that music can be used to facilitate diversionary tactics or escapism from stressors through based on *Substance Use* being theoretically categorised as a *dysfunctional coping strategy* (Coolidge et al., 2000). This is consistent with existing research that has found that engagement with music may occur in dysfunctional or maladaptive manners (Miranda and Claes, 2009; Silverman, 2020). This further indicates that music may be used to promote unhealthy listening habits or behaviour in addition to healthy or positive orientated listening behaviour (van Goethem and Sloboda, 2011; Silverman, 2020). The presence of *Substance Use* as a coping strategy is consistent with findings related to negative urgency, whereby individuals may exhibit impulsive or unrestrained behaviour during times of stress; reactions to which may include increased alcohol consumption (Corbin et al., 2013). This potentially places music listening as a means to assist these *dysfunctional* responses.

With *Age* acting as a negative predictor of some MMR subscales (and showing non-significant negative trends in others) the present results suggest that there are differences between age groups when it comes to emotional regulation. This is consistent with other research that has indicated that whilst there are not substantial differences in the extent to which different age groups accept or are aware of emotional responses, younger adults appear to have greater difficulties in regulating their emotions (Orgeta, 2009) and use music to help achieve that goal. Older adults, on the other hand, appear to have greater access to emotion regulation strategies (Orgeta, 2009). Consequently, music is only used some of the time as other strategies are available.

In general, future studies should further explore or consider the direction of causality between coping strategies and music mood regulation. In the case of this study, the theoretical basis from which coping strategies were considered was from the position that coping styles are a set of constant traits of each individual. However, if they are in fact malleable or relevant to each context, as these findings may suggest, then it stands to reason that the extent of music use as well as its importance may also change in response to different stressors. Some situations may benefit from music listening more than others and as such, music listening may inform particular coping responses to become more prevalent according to the situation.

Understanding the direction of causality more clearly would assist in clarifying whether it is coping orientations (e.g., *Positive Outlook*) that increase music’s application as a mood regulator; or use of music in mood regulation that informs subsequent coping orientations. However, the context in which studies are conducted should also be considered, as the type of event or stressor may further influence both coping orientations and music in mood regulation.

### Limitations

This study has shown limitations insofar as the application of the COPE scale may have been too context specific in this instance. Furthermore, the rescaling approach from a four-point scale to a three-point scale may have not been appropriate in hindsight due to the reduction of intermittent values. Maintaining four-points on this scale may have allowed for slightly more varied or nuanced responses and as such this should be taken into account when approaching the research model in future use.

A further limitation with this study was that respondents filled out the survey out in retrospect. Data collection only took place after the specified dates of 23rd March 2020–13th May 2020. As such, responses may not have been as authentic as they would have been had data collection taken place during the strictest lockdown period. This may be due to a memory bias, potentially Fading Affect Bias (FAB) in this instance. Existing research has indicated that there is a tendency for emotional affect to fade more over time for unpleasant events than for pleasant events (Gibbons et al., 2010). This affect begins early on after an experience (within 12 h in some instances) and may persist for months, with the FAB increasing over extended periods of time. Strong negative connotations with regards to lockdown may have been subject to such a bias when considering responses were conducted in retrospect (June–July 2020). As such, more positive coping orientations, such as *positive reframing*, may have appeared more prevalent in responses due to respondents re-orientating themselves to make their experience seem less negative. Therefore, it is important to consider the degree to which *Positive Outlook* as the most significant predictor of music in mood regulation overall is subject to the FAB.

A further general consideration to make is that this study only measured coping strategies and music mood regulation in response to lockdown within one country. Further reaching studies should be considered where possible, whereby data is gathered from respondents from different nations and regions. This may facilitate a broader understanding of coping orientations and music mood regulation in response to stressors from different cultural contexts, increasing the ecological validity of findings according to varieties in lockdown experiences between nations.

Additionally, our sample consisted of a comparatively high number of musicians (41.6%), be they amateur or professional. This was unintentional but occurred nevertheless (perhaps musicians are more likely to engage with music-based research than non-musicians). Although there has been limited findings suggesting that musicians experience emotional responses to music differently to non-musicians (Saarikallio, 2010), it should be noted that there may be a sample bias if there are in fact differences to be found between musicians and non-musicians. Alternatively, it would be interesting if future research in fact explored differences between musicians and non-musicians when it comes to employing music for the purposes of mood regulation.

## Conclusion

Implications of this study are that music has been observed to function as a mood regulator in response to lockdown. *Positive Outlook*, a factor comprising of *active coping* and *positive reframing*, has appeared to be a significant predictor of music in mood regulation, suggesting that positive mindsets and pro-active measures taken during lockdown may be an overall predictor of using music for the purposes of mood regulation. The engagement with music as a mood regulator through *Positive Outlook* is suggestive that music may be a tool used for cognitive reappraisal during lockdown. Music’s emotional qualities and ability to facilitate self-reflection and affect regulation may be encouraged by positive mindsets that seek to clarify and understand the present experience through positive or reflective engagement with music, as well as for the purposes of entertainment. However, it should be acknowledged that an alternative explanation for this could be that those with greater tendencies toward positivity may subsequently be more likely to engage in positive coping strategies, overall. Recent findings by Silverman (2020) show that healthy music use, as an indicator of healthy behaviour and well-being, was a predictor for adaptive coping strategies. This does not contradict our findings that *Substance Use* was a significant predictor of music in mood regulation, as music can also be used in unhealthy ways. It is thus plausible that certain *dysfunctional* coping strategies may increase the likelihood of music listening for purposes different than those exhibiting *Positive Outlook*. Silverman (2020) reports significantly higher ratings for unhealthy music use (compared to healthy music use) in a population of adults with substance use disorder. Whilst these MMR subscales may also be predicted by *Positive Outlook*, their occurrence alongside *Substance Use* seems to suggest that music listening may serve to assist in both ‘functional’ and *dysfunctional* coping orientations. It is not clear from this study design however, to what extent coping strategies predicate subsequent engagement with music to regulate mood.

In noticing from this, there are two seemingly antithetical tactics to promote positive engagement with music through mood regulation within this context. On the one hand, *Positive Outlook* is suggestive of pragmatic or perhaps optimistic outlooks on a stressful event, whilst *Substance Use* is indicative of escapism, and diversion (hence it’s determining of being *dysfunctional*). Whilst these two methods of coping are seemingly far apart, it is interesting that both strategies were exhibited with MMR subscales overall, as opposed to being associated with a more limited number. If it were the case, for instance, that *Substance Use* was associated with some strategies but not others, then it would be reasonable to say that specific regulatory functions of music may be associated with *dysfunctional* coping orientations, and that other regulatory functions are associated with *problem-focused* or *emotion-focused* coping orientations.

There were also findings that neither *Gender*, *Living Situation* or *Employment Status* indicated significant differences in relation to MMR subscales. Saarikallio and Erkkilä (2007) indicated that the main goals of music in mood regulation are “similar regardless of factors like age or gender” (Saarikallio and Erkkilä, 2007, p. 105). This would appear consistent within the present findings in both cases. In the case of *Gender* this is somewhat clearer as it appeared to be non-significant in our analyses. *Age*, however, did show some significance in relation to music in mood regulation. This may be explained by findings that whilst different age groups may simultaneously indicate awareness and acceptance of emotional responses, older respondents are associated with greater access to regulation strategies and clarity of emotions, as well as a greater variety of emotion regulation strategies overall (Orgeta, 2009; Puente-Martínez et al., 2021).

Additionally, we found that music in mood regulation was positively associated with MMR subscales. This was an additional validation to determine that pro-active engagement with music is associated with its use in mood regulation. However, this has raised some additional thoughts that future studies should consider whether the use of music in mood regulation is predicated by the amount of time and attention that listeners are able to give music according to their daily lives. Those with more time to listen, may have more cause to use music in mood regulation as they are able to engage for longer and with a greater variety of music, maximising music’s opportunity to function for the purposes of mood regulation as seen across the seven subscales of the MMR model in this instance.

Further implications of this study are that coping strategies are malleable in response to different stressors. This indicated that the COPE model may not be applicable when clustered into theoretical factors. On the one hand, this may be due to the hypothetical structuring of the model, and as such when applied to specific contexts, lockdown in this case, factor loadings may produce different results in accordance with the stressor. On the other hand, however, the unique context of this study is very difficult to predict. It may, for instance, be more appropriate for the COPE model to be applied when attempting to measure responses to stresses or events that seem more likely to occur in people’s lives. Also, the lack of control that individuals had over the events of the pandemic and subsequent measures may have prevented the extent to which they could mitigate the experienced stressor. This may help explain why the model did not fit in with the context it was applied to. Nevertheless, future studies that use the COPE model should also consider using factor analyses to determine the factor structure of this model. This would facilitate cross-study comparisons of the model to take place, through which researchers may be able to determine new identified factors of this model.

However, a further consideration to make is whether the direction of causality is indeed from coping orientations toward the use of music in mood regulation. If it is the case that future studies determine the use of music as a mood regulator as a predictor of coping orientations, then music listening strategies may be recommended to listeners in order to encourage particular coping orientations, such as *Positive Outlook*, that are arguably more conducive to dealing with the stressor than *Substance Use* for example. It would be interesting to include some form of content analysis if this were to be researched. Musical or lyrical themes for instance, could theoretically be associated with certain emotional effects to assist in mood regulation and thus be attributed to promote pragmatic coping solutions.

Overall, this study finds that coping orientations in response to lockdown are predictors of the subsequent use of music for the purposes of mood regulation within the United Kingdom. *Positive Reframing* and *Active Coping* form a single factor (*Positive Outlook*) which is a significant predictor of each factor from the MMR subscale except *Discharge*. *Substance Use* was a predictor of each MMR subscale. *Age* appeared to have a limited negative effect on MMR scales. These findings suggest that antithetical coping orientations can serve predictors of music in mood regulation, suggestive that both healthy and unhealthy listening habits occurred alongside lockdown measures within the United Kingdom. Future long-term studies should aim to measure how abrupt or negative life events may predispose particular coping orientations to occur and how music for mood regulation may be subsequently used. Furthermore, researchers should consider applying Experience Sampling Methodologies (ESM) to such studies, in order to gather more nuanced data points over extended periods of time, as opposed to solely relying on retrospective responses.

## Data Availability Statement

The raw data supporting the conclusions of this article will be made available by the authors, without undue reservation.

## Ethics Statement

The studies involving human participants were reviewed and approved by Arts and Humanities Ethics Committee University of York. The patients/participants provided their written informed consent to participate in this study.

## Author Contributions

NH was responsible for background research, study design, data collection, analysis, and write-up. HE provided support and structural guidance throughout the processes of study design, data collection and analyses, as well as taking an editorial role. DK contributed to statistical analyses and reporting of data. All authors contributed to the article and approved the submitted version.

## Conflict of Interest

The authors declare that the research was conducted in the absence of any commercial or financial relationships that could be construed as a potential conflict of interest.

## References

[B1] Baker J. Berenbaum H. (2007). Emotional approach and problem-focused coping: a comparison of potentially adaptive strategies. *Cogn. Emot.* 2 95–118. 10.1080/02699930600562276

[B2] Barendse M. T. Oort F. J. Timmerman M. E. (2015). Using exploratory factor analysis to determine the dimensionality of discrete responses. *Struct. Equat. Model. A Multidisc. J.* 22 87–101. 10.1080/10705511.2014.934850

[B3] BBC News (2020a). *Coronavirus**: Some Return to Work As Lockdown Eases Slightly In England. [online].* Available online at: https://www.bbc.co.uk/news/uk-52642222 (accessed May 13, 2020).

[B4] BBC News (2020b). *Coronavirus: Strict New Curbs on Life In UK Announced By PM. [online].* Available online at: https://www.bbc.co.uk/news/uk-52012432 (accessed March 24, 2020).

[B5] Cabedo-Mas A. Arriaga-Sanz C. Moliner-Miravet L. (2021). Uses and Perceptions of Music in Times of COVID-19: a Spanish Population Survey. *Front. Psychol.* 11:606180. 10.3389/fpsyg.2020.606180 33510681 PMC7835488

[B6] Carver C. (1997). You want to measure coping but your protocol’ too long: consider the brief cope. *Int. J. Behav. Med.* 4 92–100. 10.1207/s15327558ijbm0401_6 16250744

[B7] Carver C. Scheier M. Weintraub J. (1989). Assessing coping strategies: a theoretically based approach. *J. Pers. Soc. Psychol.* 56 267–283. 10.1037/0022-3514.56.2.267 2926629

[B8] Cook T. Roy A. R. K. Welker K. M. (2017). Music as an emotion regulation strategy: an examination of genres of music and their roles in emotional regulation. *Psychol. Music* 47 144–154. 10.1177/0305735617734627

[B9] Coolidge F. Segal D. Hook J. Stewart S. (2000). Personality disorders and coping among anxious older adults. *J. Anxiety Disord.* 14 157–172. 10.1016/S0887-6185(99)00046-8 10864383

[B10] Cooper C. Katona C. Orrell M. Livingston G. (2006). Coping strategies and anxiety in caregivers of people with Alzheimer’s disease: the LASER-AD study. *J. Affect. Disord.* 90 15–20. 10.1016/j.jad.2005.08.017 16337688

[B11] Corbin W. Farmer N. Nolen-Hoekesma S. (2013). Relations among stress, coping strategies, coping motives, alcohol consumption and related problems: a mediated moderation model. *Addict. Behav.* 38 1912–1919. 10.1016/j.addbeh.2012.12.005 23380486

[B12] Endler N. S. Parker J. D. A. (1990). Multidimensional assessment of coping: a critical evaluation. *J. Pers. Soc. Psychol*. 58 844–854. 10.1037/0022-3514.58.5.844 2348372

[B13] Folkman S. Lazarus R. S. (1980). An analysis of coping in a middle- aged community sample. *J. Health Soc. Behav.* 21 219–239. 10.2307/2136617 7410799

[B14] Folkman S. Lazarus R. S. (1985). If it changes it must be a process: a study of emotion and coping during three stages of a college exami- nation. *J. Pers. Soc. Psychol.* 48 150–170. 10.1037//0022-3514.48.1.150 2980281

[B15] Gibbons J. Lee S. Walker R. (2010). The fading affect bias begins within 12 hours and persists for 3 months. *Appl. Cogn. Psychol.* 25 663–672. 10.1002/acp.1738

[B16] Greb F. Scholtz W. Steffens J. (2018). Personal and situational influences on the functions of music listening. *Psychol. Music* 46 763–794. 10.1177/0305735617724883

[B17] Gustems-Carnicer J. Calderón C. (2013). Coping strategies and psychological well-being among teacher education students. *Eur. J. Psychol. Educ.* 28 1127–1140. 10.1007/s10212-012-0158-x

[B18] Karreman A. Laceulle O. Hanser W. Vingerhoets A. (2017). Effects of emotion regulation strategies on music-elicited emotions: an experimental study explaining individual differences. *Pers. Individ. Diff.* 114 36–41. 10.1016/j.paid.2017.03.059

[B19] Konečni V. (1982). “Social Interaction and musical preference,” in *The Psychology of Music*, ed. Deutsch D. (New York, NY: Academic Press), 497–516.

[B20] Krause A. Dimmock J. Rebar A. Jackson B. (2021). Music listening predicted improved life satisfaction in university students during early stages of the COVID-19 pandemic. *Front. Psychol.* 11:631033. 10.3389/fpsyg.2020.631033 33551940 PMC7855032

[B21] Krumhansl C. (1997). An exploratory study of musical emotions and psychophysiology. *Can. J. Exp. Psychol.* 51 336–352. 10.1037/1196-1961.51.4.336 9606949

[B22] Larsen R. J. (2000). Toward a science of mood regulation. *Psychol. Inq.* 11 129–141. 10.1207/S15327965PLI1103_01

[B23] Lazarus R. Folkman S. (1984). *Stress, Appraisal, and Coping.* New York, NY: Springer.

[B24] Miranda D. Claes M. (2009). Music listening, coping, peer affiliation and depression in adolescence. *Psychol. Music* 37 215–233. 10.1177/0305735608097245

[B25] Miranda D. Gaudreau P. Morizot J. (2010). Blue notes: coping by music listening predicts neuroticism changes in adolescence. *Psychol. Aesthet. Creativ. Arts* 4 247–253. 10.1037/a0019496

[B26] Orgeta V. (2009). Specificity of age differences in emotion regulation. *Aging Ment. Health* 13 818–826. 10.1080/13607860902989661 19888702

[B27] Puente-Martínez A. Prizmic-Larsen Z. Larsen R. J. Ubillos-Landa S. Páez-Rovira D. (2021). Age differences in emotion regulation during ongoing affective life: a naturalistic experience sampling study. *Am. Psychol. Assoc.* 57 126–138. 10.1037/dev0001138 33382328

[B28] Rosseel Y. (2012). lavaan: an r package for structural equation modeling. *J. Stat. Softw.* 48 1–36. 10.3389/fpsyg.2014.01521 25601849 PMC4283449

[B29] Roy M. Mailhot J.-P. Gosselin N. Paquette S. Peretz I. (2009). Modulation of the startle reflex by pleasant and unpleasant music. *Int. J. Psychophysiol.* 71 37–42. 10.1016/j.ijpsycho.2008.07.010 18725255

[B30] Saarikallio S. (2008). Music in mood regulation: initial scale development. *Music. Sci.* 12 291–309. 10.1177/102986490801200206

[B31] Saarikallio S. (2010). Music as emotional self-regulation throughout adulthood. *Psychol. Music* 39 307–327. 10.1177/0305735610374894

[B32] Saarikallio S. Erkkilä J. (2007). The role of music in adolescents’ mood regulation. *Psychol. Music* 35 88–109. 10.1177/0305735607068889

[B33] Silverman M. (2020). Music-based affect regulation and unhealthy music use explain coping stratigies in adults with mental health conditions. *Commun. Ment. Health J.* 56 939–946. 10.1007/s10597-020-00560-4 31997124

[B34] Stewart J. Garrido S. Hense C. McFerran K. (2019). Music use for mood regulation: self-awareness and conscious listening choices in young people with tendencies to depression. *Front. Psychol.* 10:1199. 10.3389/fpsyg.2019.01199 31178806 PMC6542982

[B35] Ter Bogt T. Mulder J. Raaijmakers Q. Gabhainn S. (2010). Moved by music: a typology of music listeners. *Psychol. Music* 39 147–163. 10.1177/0305735610370223

[B36] Thayer R. E. Newman J. R. McClain T. M. (1994). Self-regulation of mood: strategies for changing a bad mood, raising energy, and reducing tension. *J. Pers. Soc. Psychol.* 67 910–925. 10.1037//0022-3514.67.5.910 7983582

[B37] Tobin D. L. Holroyd K. A. Reynolds R. V. Wigal J. K. (1989). The hierarchical factor structure of the coping strategies inventory. *Cogn. Ther. Res.* 13 343–361.

[B38] van Goethem A. Sloboda J. (2011). The functions of music for affect regulation. *Music. Sci.* 15 208–228. 10.1177/1029864911401174

