# Supplementary material for: Music in Mood Regulation and Coping Orientations in Response to COVID-19 Lockdown Measures Within the United Kingdom
Source: Front Psychol. 2021 May 19;12:647879. doi: 10.3389/fpsyg.2021.647879 (PMC8170082; doi:10.3389/fpsyg.2021.647879)
Supplement: Supplementary file 1 [file Data_Sheet_1.pdf]

Supplementary Material

Appendix A

Survey

Q1 During lockdown, how many hours each day did you listen to music on average?

Q2 Is this more or less than before lockdown?

- More
- Less
- The same
- Don't Know

Q3 What media have you primarily used to listen to music? (Tick all that apply)

- Radio
- Audio streaming Services (Spotify, Apple Music etc)
- Physical Formats (CDs, Vinyl etc)
- Video streaming services (for example YouTube)
- Other

Q4 MMR Items

These questions will ask you about your engagement with music during lockdown. Please respond from this perspective rather than music listening more generally.

When thinking about your time in lockdown, to what extent do you agree with the following statements?

Item 1: When busy around the house and no one else has been present, I have liked to have some music on the background

Item 2: When going out for daily exercise or a change of scene, I listened to music to get myself in the right mood

Item 3: I’ve listened to music to make cleaning and other housework more pleasant

Item 4: I have been putting background music on to make the atmosphere more pleasant

Item 5: When tired, I have rested by listening to music

Item 6: Listening to music hasn’t help me to relax

Item 7: I’ve listened to music to perk up after a rough day

Item 8: When I’ve been exhausted, I have listened to music to perk up

Item 9: When I’ve been exhausted, music has given me new energy

Item 10: I have listened to music to get breathing space in the middle of a busy day

Item 11: Listening to music has helped me to relax

Item 12: I have felt fantastic putting my soul fully into music

Item 13: Music has been offering me magnificent experiences

Item 14: Music has been offering me unforgettable moments

Item 15: Music has not evoked strong emotional experiences in me

Item 16: I have wanted to listen to music that evokes feelings in me

Item 17: I have wanted to feel music in my whole body

Item 18: Sometimes music has felt so great that I have gotten goose bumps (in a positive sense)

Item 19: When stressful thoughts have kept going around in my head, I have listened to music to get them off my mind

Item 20: For me, music has been a way to forget about my worries

Item 21: Listening to music has helped to block out disturbing factors from my mind

Item 22: When I’ve been feeling bad, I try to get myself in a better mood by engaging in a pleasant, music-related activity

Item 23: I can’t push my worries aside with the help of music

Item 24: When I have been angry, I vent that anger by listening to music that expresses my anger

Item 25: When everything has felt miserable, I've started listening to music that expresses these feelings

Item 26: When I’ve been angry with someone, I have listened to music that expresses my anger

Item 27: When I’ve been really angry, I have felt like listening to some angry music

Item 28: When I’ve been angry, I have almost never listened to angry music

Item 29: When everything has felt bad, it has helped me to listen to music that expresses my bad feelings

Item 30: Music has helped me to work through this hard experience

Item 31: Music has helped me to understand different feelings in myself

Item 32: Listening to music has taken me back and gets me thinking about different things that have happened to me

Item 33: Music has inspired me to think about important issues

Item 34: When I’ve been feeling distressed by the current situation, music has helped me to clarify my feelings

Item 35: When something is troubling me, I have found solace in music

Item 36: I have listened to music to find solace when worries overwhelm me

Item 37: Listening to music hasn’t comforted me in my sorrows

Item 38: When everything has felt bad, music understands and comforts me

Item 39: Music is like a friend who understands my worries

Item 40: When I’ve been feeling sad, listening to music comforts me

Q 5 COPE Items

When thinking about your experiences under lockdown, how well do these statements apply to you?

Item 1: I’ve been concentrating my efforts on doing my best to manage the situation

Item 2: I’ve been taking action to try to make the situation better

Item 3: I’ve been trying to come up with a strategy about what to do

Item 4: I’ve been thinking hard about what steps to take

Item 5: I’ve been trying to see it in a different light to make it seem more positive

Item 6: I’ve been looking for something good in what is happening

Item 7: I’ve been accepting the reality of the fact that this has happened

Item 8: I’ve been learning to live with it

Item 9: I’ve been making jokes about it

Item 10: I’ve been making fun of the situation

Item 11: I’ve been trying to find comfort in my religion or spiritual beliefs

Item 12: I’ve been praying or meditating

Item 13: I’ve been getting emotional support from others

Item 14: I’ve been getting comfort from and understanding from someone

Item 15: I’ve been trying to get advice or help from others about what to do

Item 16: I’ve been getting help and advice from other people

Item 17: I’ve been turning to work or other activities to take my mind off things

Item 18: I've been doing something to think about the situation less, such as watching movies, TV, reading or sleeping

Item 19: I've been saying to myself "this isn't real"

Item 20: I've been refusing to believe that it has happened

Item 21: I've been saying things to let my unpleasant feelings escape

Item 22: I've been expressing my negative feelings

Item 23: I've been using alcohol or other drugs to make myself feel better

Item 24: I've been using alcohol or other drugs to help me get through the situation

Item 25: I've been giving up trying to deal with it

Item 26: I've been giving up the attempt to cope

Item 27: I've been criticising myself

Item 28: I've been blaming myself for the things that have happened

Q6 Which of the following best describes your situation?

- The UK has been my primary residence for the majority or all of my life
- Although not being raised in the UK, I have lived here for 4 or more years and would now consider it to be my permanent country of residence
- I moved to the UK within the last 4 years and plan to stay for the foreseeable future
- I moved to the UK within the last 4 years and intend on moving back to my home country or elsewhere
- Other

Q7 Under what living circumstance have you spent lockdown?

- Alone
- Within a family unit
- With one other person
- Within a house-share
- Other

Q8 What best describes your current employment status?

- Employed full-time and able to work
- Employed part-time and able to work
- Furloughed or otherwise unable to work (but still employed)
- Unemployed
- Retired
- Other

Q9 What is your age in years?

Q10 What is your gender

- Male
- Female
- Prefer not to say
- Other

Q11 Which best describes your musical background?

- Non-musician
- Amateur musician
- Higher level musician
- Professional musician

Q12 What is your highest attained level of education?

- GCSEs
- A-Levels
- Undergraduate degree
- Master's degree
- PhD

Q13 Do you have any comments about the survey?

Appendix B

Factor Scores of MMR

MMR Item	Factor 1	Factor 2	Factor 3	Factor 4	Factor 5	Factor 6	Factor 7
Entertainment: Background	.744						
Entertainment: Going Out	.774						
Entertainment: Housework	.669						
Entertainment: Atmosphere	.596						
Revival: Tired		.759					
Revival: Perk Up After Rough Day		.790					
Revival: Perk Up Exhausted		.815					
Revival: New Energy		.642					
Revival: Breathing Space		.832					
Revival: Relax		.622					
Revival: Hasn't helped relax (*reversed item*)		.305					
Strong Sensation: Soul into Music			.982				
Strong Sensation: Magnificent Experiences			.842				
Strong Sensation: Unforgettable Moments			.788				
Strong Sensation: Evoked Strong Feelings			.708				
Strong Sensation: Whole Body			.886				
Strong Sensation: Goosebumps			.843				
			.389				

Strong Sensation: Not Evoked strong feelings (*reversed item)*				
Diversion: Stressful Thoughts	.986			
Diversion: Forget About Worries	.833			
Diversion: Block Out Disturbing Factors	.673			
Diversion: Better Mood by Listening	.635			
Diversion: Can't push worries aside (*reversed item*)	.372			
Discharge: Venting Anger		1.180		
Discharge: Miserable Expression		.895		
Discharge: Express Anger at Someone		1.090		
Discharge: Angry Mood Angry Music		1.142		
Discharge: Music Expresses Bad Feelings		1.156		
Discharge: Never listens to angry music (*reversed item*)		.727		
Mental Work: Music Helped Through Experience			.813	
Mental Work: Understand Different Feelings			.851	
Mental Work: Thinking About Past Experiences			.613	
Mental Work: Thinking About Important Issues			.750	
Mental Work: Clarify Feelings When Distressed			.885	
Solace: Finding Solace When Troubled				.800
Solace: Finding Solace When Overwhelmed				.940

Solace: Understanding and Comfort	.861
Solace: Music Like Friend	.896
Solace: Music Comforts Me	.811
Solace: Music has not comforted me (*reversed item*)	.686
